# Recent Progress in Otology

**Published:** 1877-11

**Authors:** 


Recent Progress in Otology.—*The Nasal Douche and the Ear.*—Inflammations of the middle ear, both catarrhal and purulent, have been frequently reported in the last few years as the direct result of the use of the nasal douche, and many unreported cases have occurred in the practice of those who have seen much of aural disease since the extensive use—and abuse—of Weber's apparatus. The reckless employment of the instrument without advice, and with almost every imaginable fluid, and for any and every condition af the naso-pharyngeal cavity, including even fancied collections of dust, was, undoubtedly, the cause of some of the earlier accidents to the ear; but further experience has shown that even with all the cautions which the physican can give it will occasionally happen that any fluid introduced into the naso-pharyngeal cavity with force, be it by the douche, or the nasal or pharyngeal syringe, may find its way into the eustachian tube, and set up an inflammation of the middle ear. To diminish the risk of this accident, as far as possible, the following cautions are now generally recognized as necessary to be observed with the use of either the douche or syringe: The water should be lukewarm, and contain a small quantity of salt, to make its specific gravity as nearly equal to that of the blood as possible; any great pressure should be avoided; the act of swallowing, or any other movement of the pharyngeal muscles which tends to open the eustachian tube, should be carefully prevented by the patient; blowing of the nose and sneezing, for some little time after the operation, should also be avoided. In spite of these cautions occasional accidents to the ear still occur, even when the physician himself has superintended the application of the fluid; and Buck has recently reported cases of inflammation of the middle ear from the simple snuffing up of salt water into the nose, no syringe being used. The argument that has been used, namely, that the ear disease, after the use of the fluid, is a mere coincidence, and not the result of the operation, is sufficiently refuted by the clinical histories of many of the cases; for the passage of the liquid toward the ear is distinctly felt by the patient at the time, and is followed immediately by the pain, which continues through the course of the disease.

These being the facts, as learned by experience, some writers have urged that the use of fluids in any quantity in the naso-pharynx should be entirely discarded, and that the objects of cleansing the cavity and of making applications to it should be done by sponge or brush. Others, recognizing the great value of douching for certain diseases, especially ozæna, are endeavoring to add to the cautions already observed, such others as shall wholly prevent the possibility of the fluid reachthe ear. Zaufal considers the use of the douche as absolutely necessary in ozæna and certain ear disease dependent on disease of the nasal mucous membrane, and would prevent the entrance of fluid into the eustachian tubes, by a mechanical closure of those tubes. He has seen demonstrated, that pressure of the soft palate towards the orifices of the tubes, has the effect of raising the floor of the eustachian tube so high that the tubal cartilage is pressed back, and the floor of the tube is pressed against the cartilage-hook, and the orifice is tightly closed. Accordingly, when he wishes to apply the douche, he stands behind the patient, and with two fingers closes the tubes by a firm pressure of the soft palate against them.

Frankel, while acknowledging the effectiveness of this expedient of Zaufal's, considers that it is not always, or even generally, applicable, but thinks that the same closure of the tubes can be produced by the phonation of the non-nasal vowels. His practice is as follows: before using any applications he satisfies himself that both nostrils are free enough to allow the exit of fluid, and this is readily accomplished by closing first one nostril and then the other, while he is listening to the breathing of the patient. Instead of a Weber's douche, he uses the rubber bulb syringe of Michel (the Davidson syringe would be a good substitute), which enables the patient to check the current when necessary. The patient is then directed, before compressing the bulb—that is, beginning the douche—to pronounce the vowel o, and to continue this phonation till after the douching is over, thus closing the palate against the posterior wall of the pharynx and the eustachian tubes, by contraction of the levator palati muscle. The advantage of this use of phonation over the former method of douching is, that the naso-pharyngeal isthmus is already closed before the fluid enters the nose, instead of closing by reflex irritation after the entrance of the fluid.

In addition to the cautions already given, these expedients of Zaufal and Frankel can well be borne in mind in prescribing any form of the douche—the former when the surgeon himself makes the application, the latter when it is intrusted to an intelligent patient. But, unfortunately, the intelligence of patients, in a surgical sense, cannot be depended upon, and the question of whether the benefit from the douche more than counterbalances the slight risk to the ear, must be left to the decision of the prescribing physician. Certainly, the cases of ear disease which have been reported, should lead to a careful consideration of each individual nose, and should check the reckless and indiscriminate use of the douche.

*Rupture of the Membrana Tympani by Hanging.*—Zaufal explains the well-known fact that the membrana tympani is often found ruptured in persons who have died by hanging, as follows: from the compression of the neck the tongue is forced upwards against the soft palate, and this, in turn, presses the floor of the eustachian against its roof, closing the tube. This closure taking place very rapidly, the air within the tympanum is condensed, and ruptures through the membrane.

*Syphilitic Disease of the Labyrinth.*—Comparatively few and imperfect observations of the histiological changes of the labyrinth in syphilis have been published, and the following by Moos is of special interest: A man, aged forty-four, after seven years of constitutional syphilis, complained, with other symptoms of tertiary disease, of "frightful" subjective noises of all varieties in the ears, and occasional attacks of dizziness. The hearing was perfect, and inspection showed nothing. Three months after, the perspection of higher tones and the ticking of a watch was diminished, but conversation was well heard. In six weeks more the perception of the voice was seriously affected, and within a week the deafness was very extreme. Death from bronchitis followed eight days after. Dissection of one ear showed sclerosis of the petros bone; the external and middle ear were normal throughout, and the only changes were in the labyrinth. The periosteum of the vestibule was thickened, and the base of the stapes immovable; the connective tissue of the vestibule hyperplastic, and with small-cell infiltration; the periosteum of the lamina spiralis ossea and the different zones of the lamina spiralis membranacea, more especially the pillars and arches of corti, likewise inflated; the acoustic nerve was normal.

These microscopic appearances correspond, Moos says, with Virchow's and Wagner's descriptions of the earlier stages of syphilitic new growths, and he considers that the pathological process in the ear started from the periosteal connective tissue of the vestibule, and extended by contiguity to the cochlea, of which the zones were somewhat unequally affected. The primary disease of the vestibule, he thinks, was most probably caused by the extension of a syphilitic inflammation of the connective substance of the bones and the periosteum of the skull to the labarynth, the first appearance of the ear symptoms being simultaneous with the characteristic syphilitic headache. The earlier ear symptoms, subjective noises, and vertigo, were caused by the periosteal irritation in the vestibule; the later, rapidly-increasing deafness, etc., by the anchilosis of the stapes and the infiltration of the cochlea, this latter especially being sufficient, by its pressure, to obliterate the functions of the nervous structures.

The earlier pathological changes, that is, the small-cell infiltration, Moos thinks would lead one to expect in some cases a cure from appropriate medication, provided always that atrophy, caseous degeneration or ulceration, had not set in. The latter changes, however, the hyperplastic, would be beyond hope.

From the symptoms in this case and from his previous experience, Moos agrees with Hinton that an early and very rapid loss of hearing is an important symptom for the diagnosis of syphilitic ear disease. An early diminution of the perception through the bones he has also observed. Extreme deafness from syphilis he has never been able to affect by any therapeutic means, but the true "nervous" syphilitic cases in the very earliest stages he has found amenable to treatment.

*Extension of Inflammation from the Brain to the Tympanum.*—Inflammation of the brain, showing itself as meningitis, encephalitis, or thrombosis from an extension of an inflammatory process in the ear, is by no means uncommon, and the avenues by which such an extension takes place have been carefully studied and are quite well understood. The reversed process, however, an extension of inflammation from the brain through any of these avenues to the ear, is very rare, almost the only instances confirmed by post-mortem being cases of cerebro-spinal meningitis, in which the membranous labyrinth, or the tympanum, or both, were found in various stages of inflammation. Berndgen reports a case of interest, both as an instance of secondary inflammation of the tympanum dependent on brain disease and of an unusual avenue of communication between these two organs. A strong man, aged twenty-one, complained of severe pain in the left ear, and presented all the appearances of an inflammation of the tympanum, serous infiltration, and marked redness of the membrana tympani, etc. Under the use of leeches and the air-douche this rapidly improved, and he resumed work, but returned after a few days on account of an attack of severe dizziness, which it was feared depended on extension of the inflammation to the brain. The inflammation of the ear had much diminished. The dizziness soon passed off, but eight days after he had severe temporal headache, which was followed in four days by sopor, clonic spasms, and death, with the symptoms of paralysis of the brain. The autopsy showed an abscess, the size of a pigeon's egg, in the substance of the left cerebellum, which had ruptured through the dura mater, and a little pus was lying external to this membrane. The dura mater itself on the left side was hyperæmic and thickened, and this was more particularly the case in the immediate neighborhood of the fissura petrosquamosa, which in this case had never closed, but remained in the state in which it is generally found in children when it forms a direct communication between the cavity of the skull and that of the tympanum. The mucous membrane of the tympanum showed only slight redness, swelling, and a little catarrhal secretion. The roof of the tympanum and the dura mater covering it were intact. The mitral valves of the heart showed marked thickening. Other organs normal.

The condition of the abscess, its size, and the thickening of the dura mater, together with the slight disease of the tympanum, leave no doubt that the brain disease was primary and the tympanic disease secondary, or more correctly, that the encephalitis occurred first, this extended to the dura mater, and then through the abnormally open fissura petrosquamosa to the tympanum. This communication between the encephalon and tympanum, which must be referred to an arrest of development, adds another to the numerous channels for the extension of Inflammation from one organ to the other.

The case of Berndgen is another instance of the insidious ourse of abscess of the brain.—*Boston Medical and Surgical Journal.*

